# Supplementary material for: Structural insights into NDH-1 mediated cyclic electron transfer
Source: Nat Commun. 2020 Feb 14;11:888. doi: 10.1038/s41467-020-14732-z (PMC7021789; doi:10.1038/s41467-020-14732-z)
Supplement: Supplementary file 1 — Supplementary Information [file 41467_2020_14732_MOESM1_ESM.pdf]

Supplementary Information

Structural insights into NDH-1 mediated cyclic electron transfer

C. Zhang, J. Shuai, Z. Ran et al.

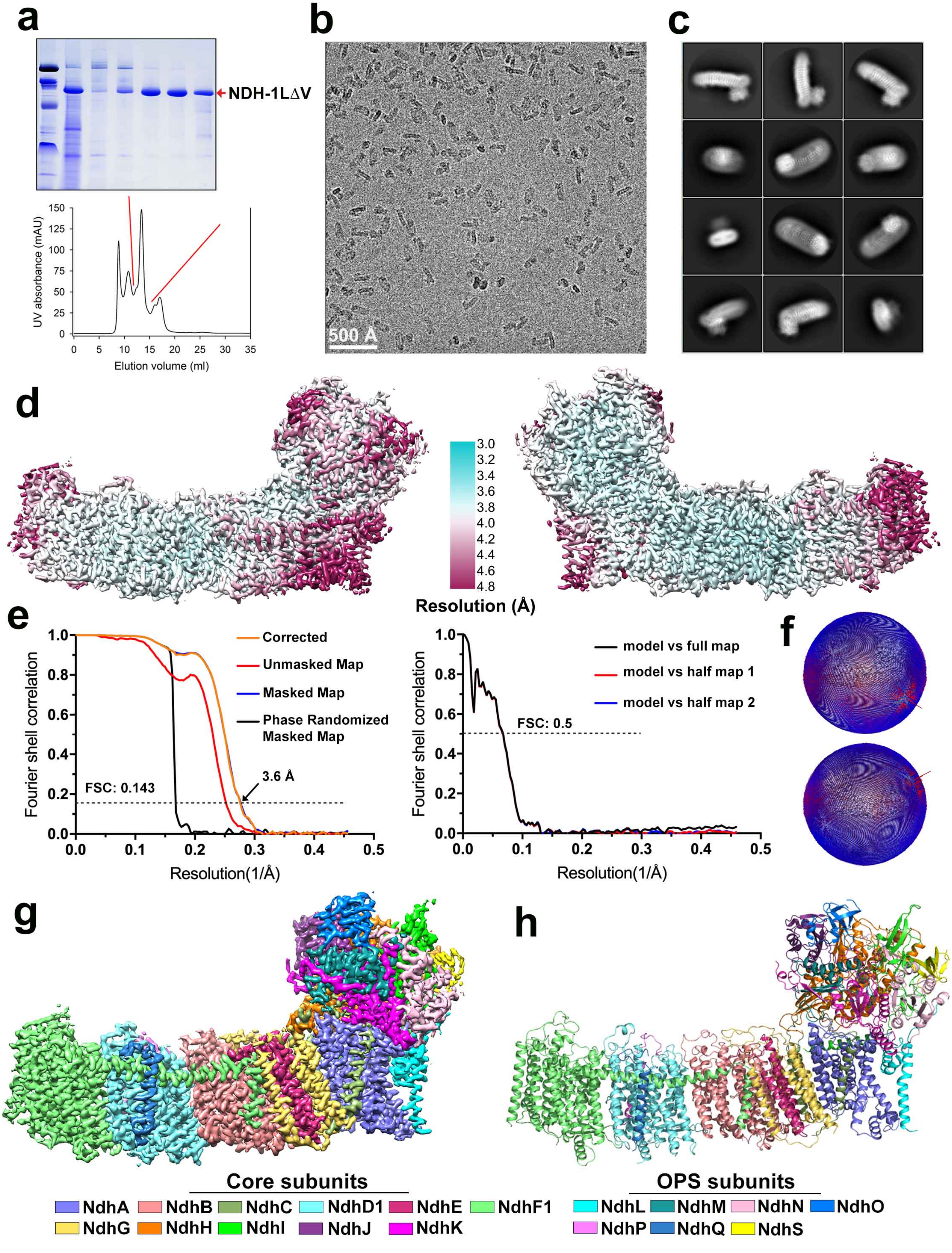

Supplementary Figure 1

Supplementary Figure 1

Purification and cryo-EM analysis of the *T. elongatus* NDH-1LΔV complex

(a) Blue native PAGE gel of the NDH-1LΔV complex purified from *T. elongatus* using Ni^{2+} affinity chromatography (NAC) followed by size-exclusion chromatography (SEC). Position of NDH-1LΔV in the gel is indicated. **(b)** A representative cryo-EM micrograph of *T. elongatus* NDH-1LΔV. **(c)** Representative 2D class averages of the NDH-1LΔV particles were obtained from reference-free classification. **(d)** Local resolution of the final cryo-EM density map estimated by RELION 3. The resolution is color-coded for different regions of NDH-1LΔV. **(e)** The fourier shell correlation (FSC) curves of the final EM density map. The resolution was determined to an average of 3.6 Å with a cut-off value of 0.143 (left panel). The FSC curves were calculated between the refined atomic model and the half map used for refinement (red), the other half map (blue) and the full map (black) (right panel). The cut-off value of FSC is 0.5. **(f)** Angular distributions of particles for the final reconstruction of *T. elongatus* NDH-1LΔV. Red cylinders represent more particles on these orientations. Heights of cylinders indicate the relative numbers of particles. **(g)** The cryo-EM reconstruction of the *T. elongatus* NDH-1LΔV complex. The cryo-EM density map is segmented by subunits of NDH-1LΔV and the color scheme of the subunits is shown below. **(h)** Overview of the NDH-1LΔV complex in cartoon representation.

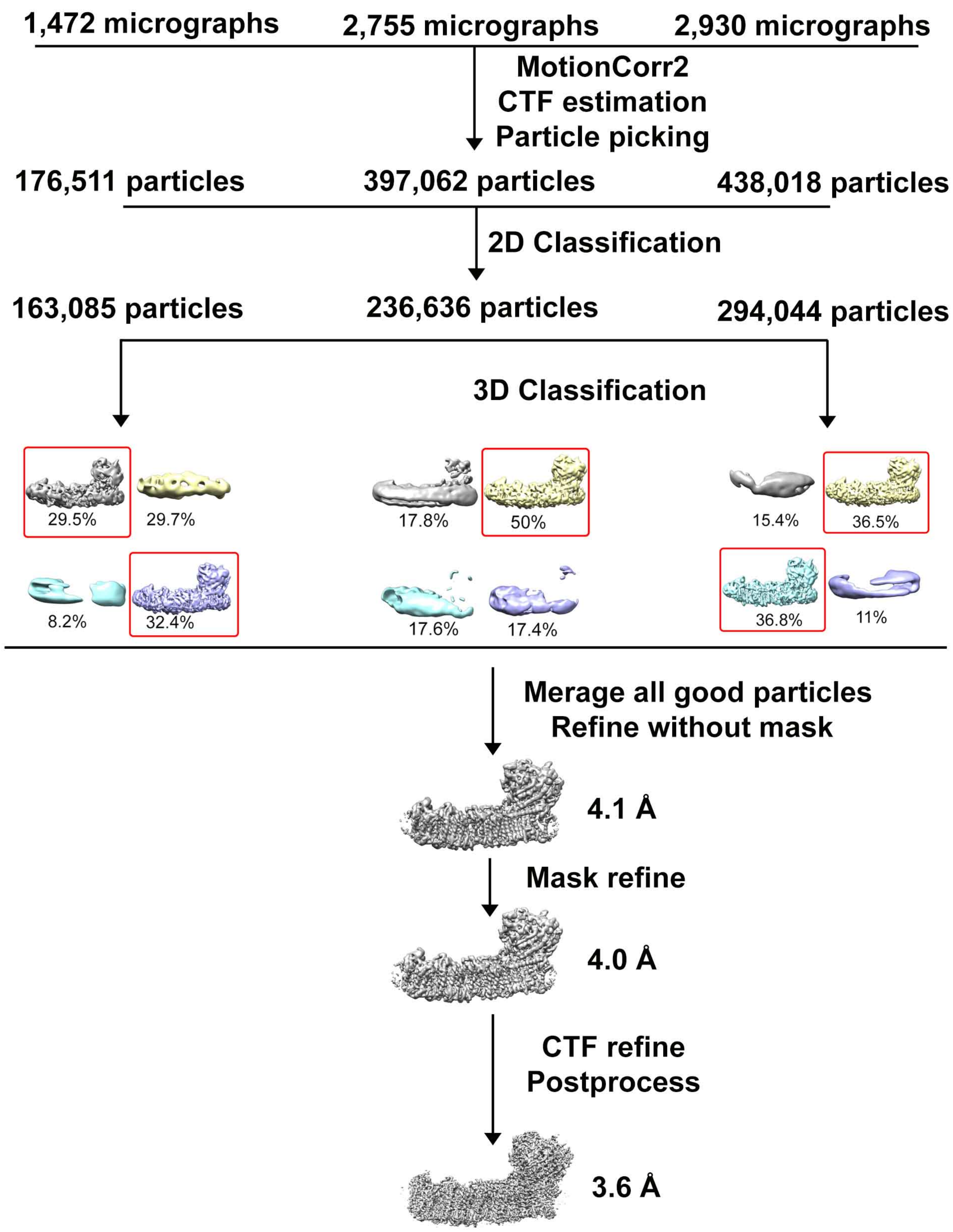

Supplementary Figure 2

Supplementary Figure 2

Flow chart for the cryo-EM data processing and structure determination of the *T. elongatus* NDH-1LΔV complex

Refer to Materials and Methods for details. The final reconstruction has an average resolution of 3.6 Å. All the images in this figure are created by Chimera.

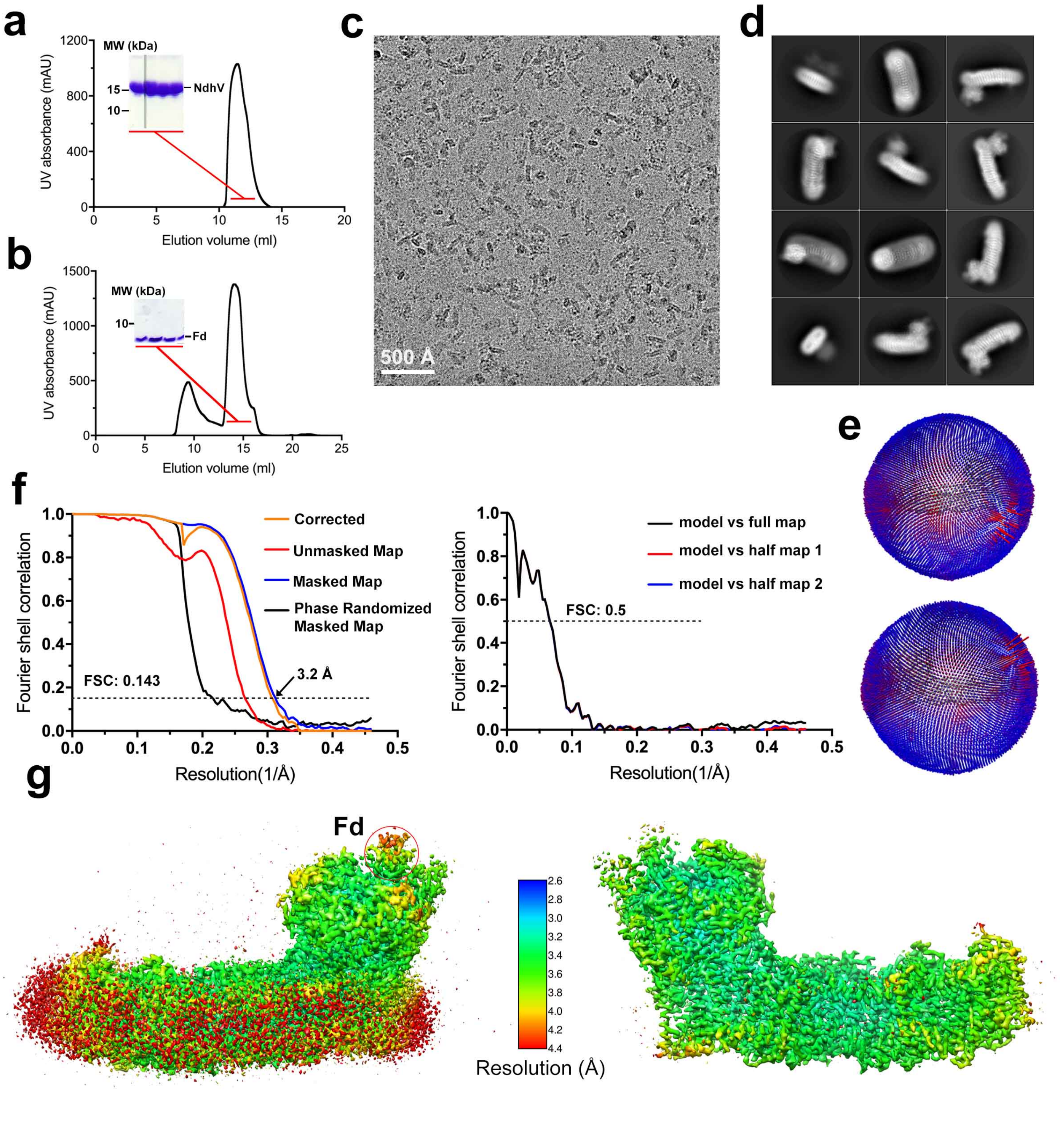

Supplementary Figure 3

Supplementary Figure 3

***In vitro* reconstitution and cryo-EM analysis of the *T. elongatus* Fd-NDH-1L complex**

(a) SEC analysis of purified *T. elongatus* NdhV. Inset is the SDS-PAGE gel showing the purity of NdhV at the peak position on the SEC profile. **(b)** SEC analysis of purified *T. elongatus* Fd. Inset is the SDS-PAGE gel showing the purity of Fd at the peak position on the SEC profile. **(c)** A representative cryo-EM micrograph of the *T. elongatus* Fd-NDH-1L complex. **(d)** Representative 2D class averages of Fd-NDH-1L complex particles were obtained from reference-free classification. **(e)** Angular distributions of particles for the final reconstruction of the *T. elongatus* Fd-NDH-1L complex. Red cylinders represent more particles on these orientations. Heights of cylinders indicate the relative numbers of particles. **(f)** The FSC curves of the final EM density map. The resolution was determined to an average of 3.2 Å with a cut-off value of 0.143 (left panel). The FSC curves were calculated between the refined atomic model and the half map used for refinement (red), the other half map (blue) and the full map (black) (right panel). The cut-off value of FSC is 0.5. **(g)** Local resolution of the final cryo-EM map estimated by RELION 3. The resolution is color-coded for different regions of the Fd-NDH-1L complex. The left panel is drawn at a lower threshold to better show the EM density of Fd. Extra densities in the membrane domain are those of the detergent (digitonin) used in the *in vitro* reconstitution of the Fd-NDH-1L complex.

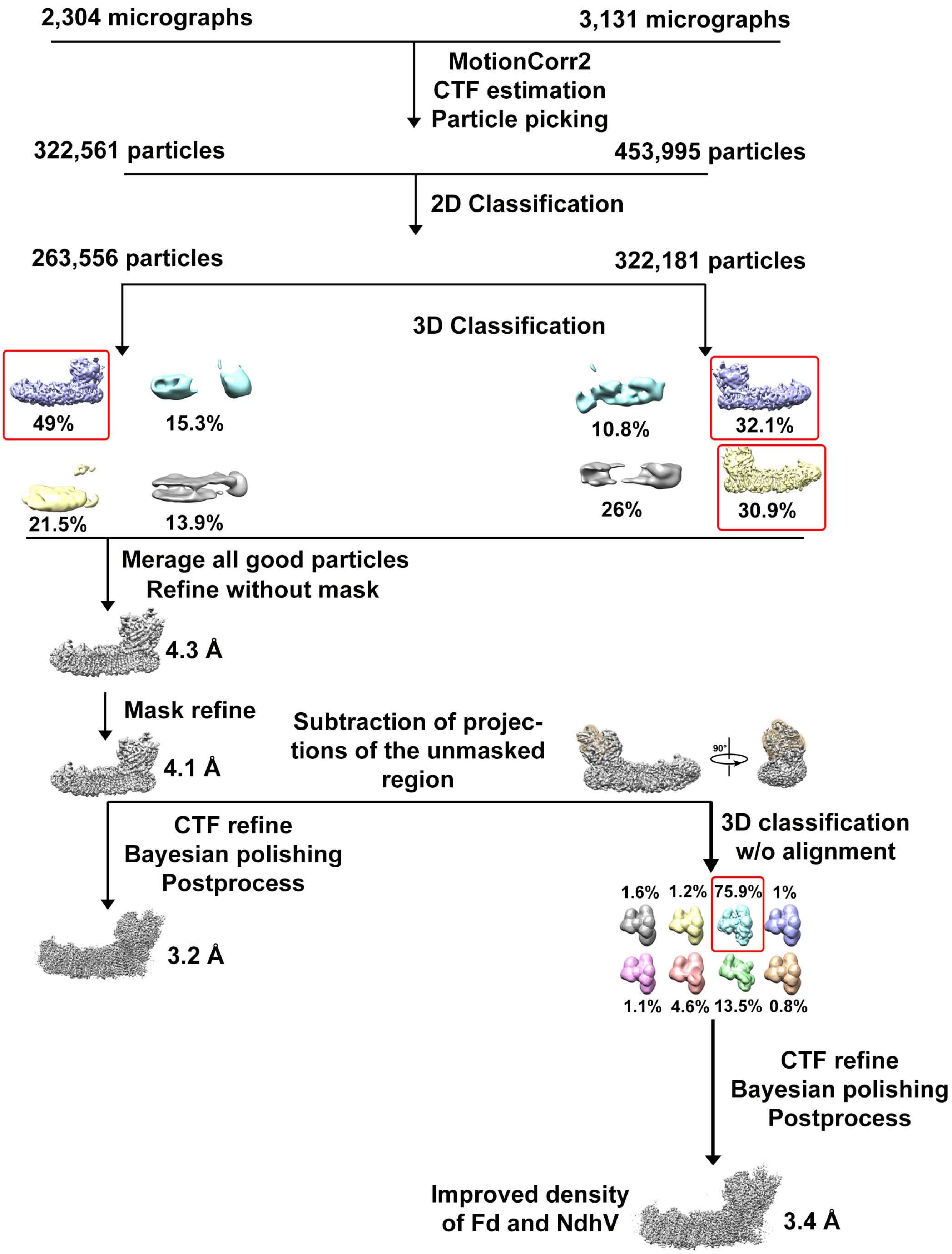

Supplementary Figure 4

Supplementary Figure 4

Flow chart for the cryo-EM data processing and structure determination of the *T. elongatus* Fd-NDH-1L complex

The final reconstruction has an average resolution of 3.2 Å. All the images in this figure are created by Chimera.

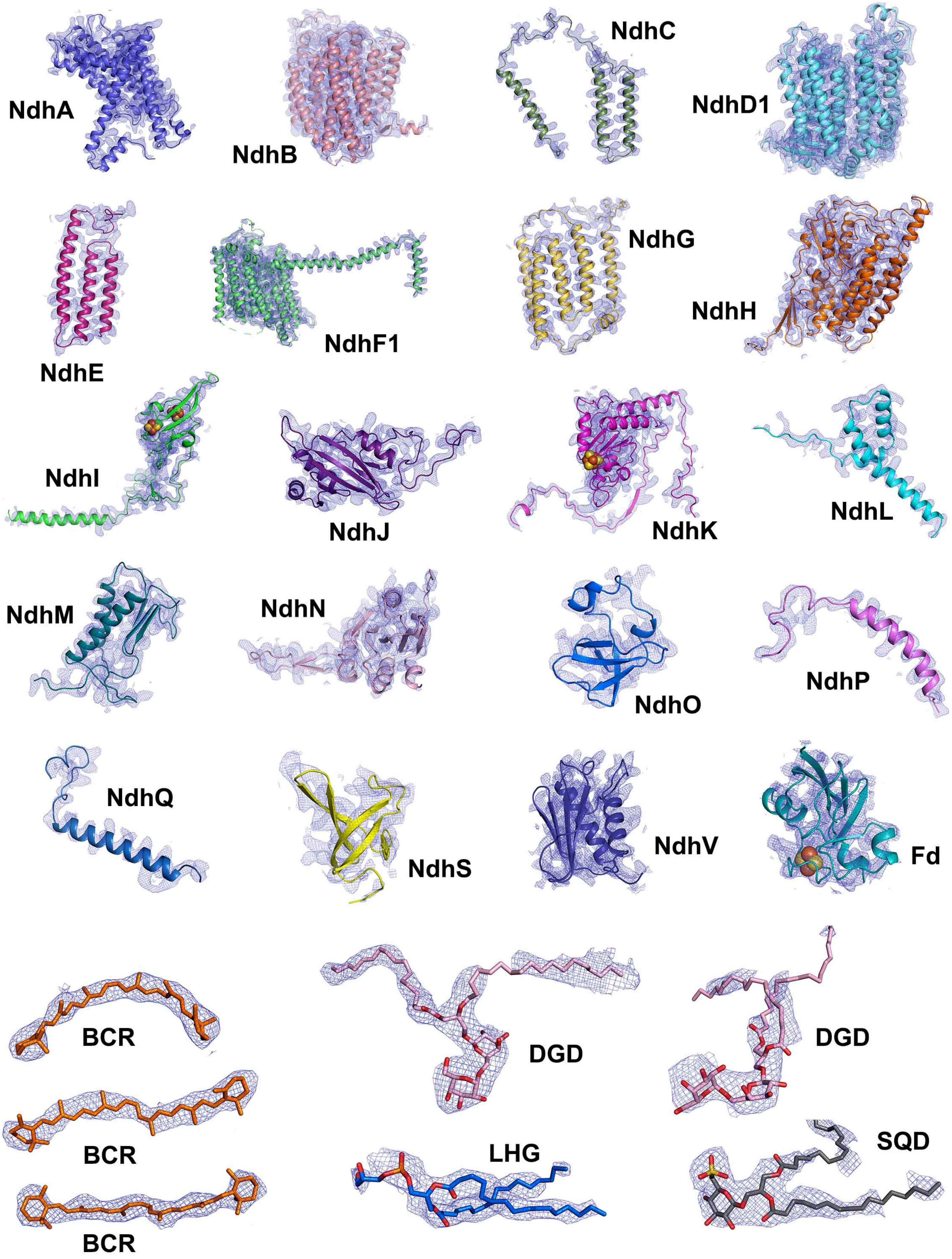

Supplementary Figure 5

Supplementary Figure 5

A gallery of cryo-EM density maps of NDH-1L subunits, Fd, and representative cofactors in the Fd-NDH-1L complex

NDH-1L subunits, Fd and representative cofactors are labeled and shown in cartoon representation. BCR, β-carotene; LHG, dipalmitoylphosphatidylglycerol; DGD, digalactosyldiacylglycerol; SQD, sulfoquinovosyldiacylglycerol.

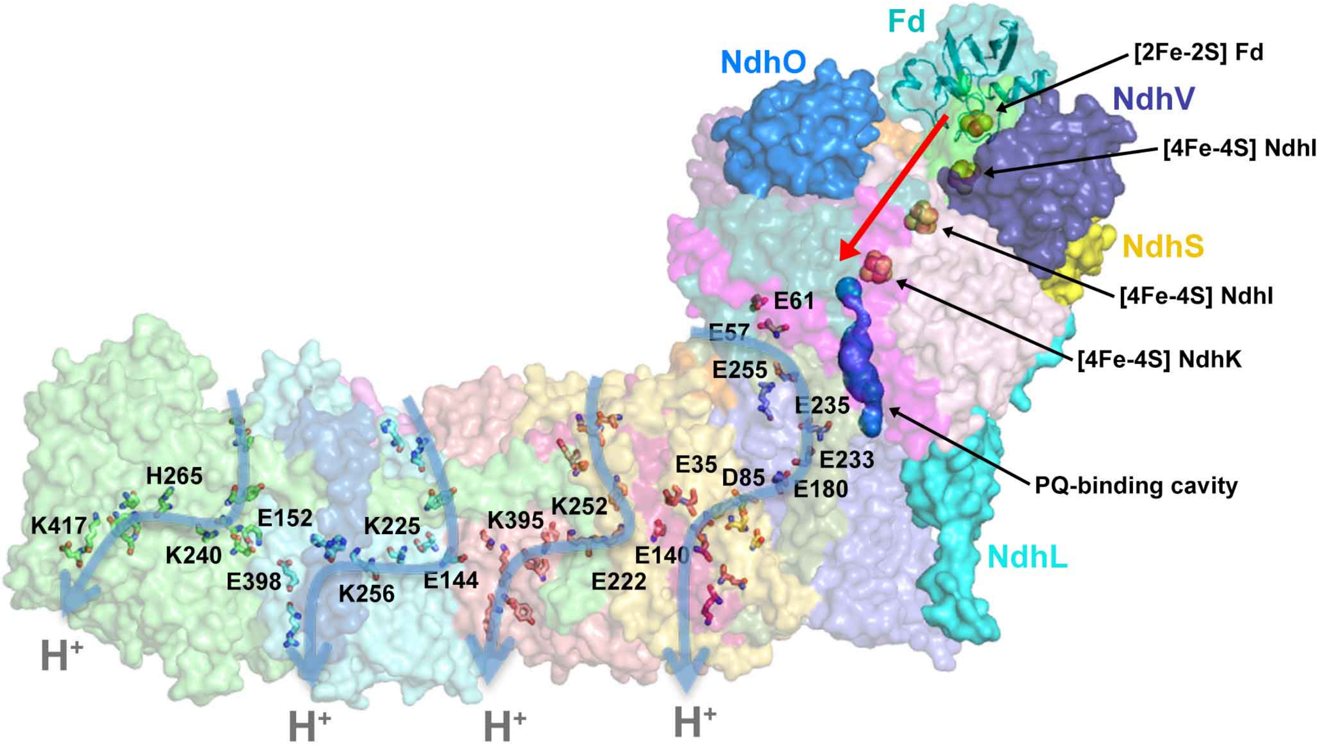

Supplementary Figure 6

Supplementary Figure 6

Putative proton-translocation pathways of the *T. elongatus* Fd-NDH-1L complex

Arrangement of redox centers, putative PQ-binding cavity and four putative proton pumping pathways. The Fd-NDH-1L complex is in surface representation and colored as in **Fig. 1b**. Ion-sulfur clusters are shown as spheres and the electron transfer pathway is denoted by a red arrow. Putative proton translocation pathways are indicated by blue arrows. The PQ-binding cavity (blue) in NDH-1L was predicted using CAVER 3.0. Key charged and polar residues in proton pumping pathways are labeled and shown in stick representation.

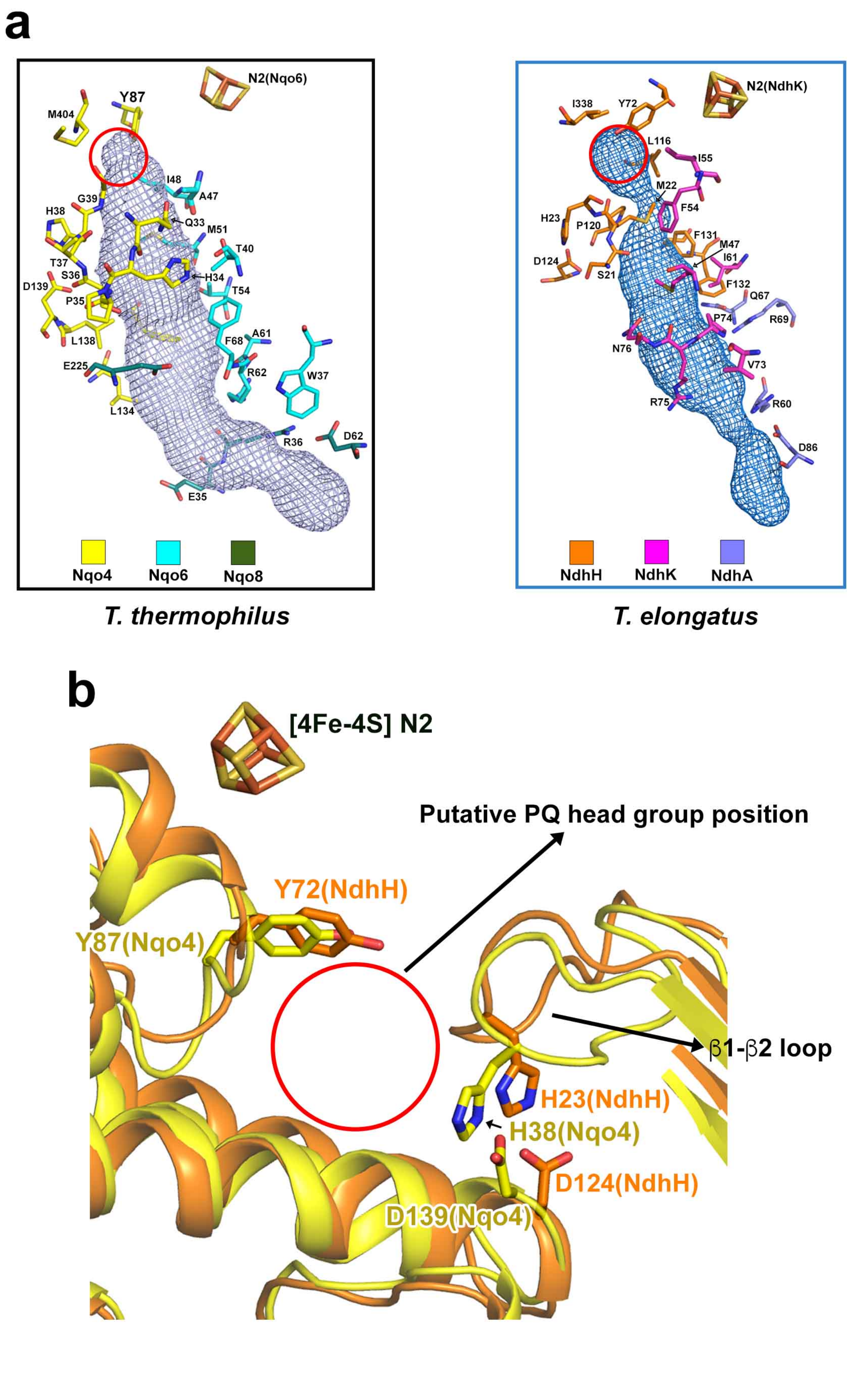

Supplementary Figure 7

Supplementary Figure 7

Active conformation of the quinone-binding cavity in the Fd-NDH-1L complex

(a) Comparison of structural environments around cluster N2 and the quinone-binding cavity in the structures of *T. thermophilus* Complex I (PDB 4HEA), and *T. elongatus* NDH-1L. The quinone-binding cavity was predicted using CAVER 3.0. Key residues for quinone binding are shown as sticks and colored as indicated below. **(b)** Close-up view of the putative quinone head group-binding site with subunits colored as in (a). Three key residues Tyr72, His23, Asp124 of *T. elongatus* NDH-1L at the putative quinone head group-binding site adopt similar conformations as equivalent residues in *T. thermophilus* Complex I, suggestive of an active state of NDH-1L that is ready for quinone binding.

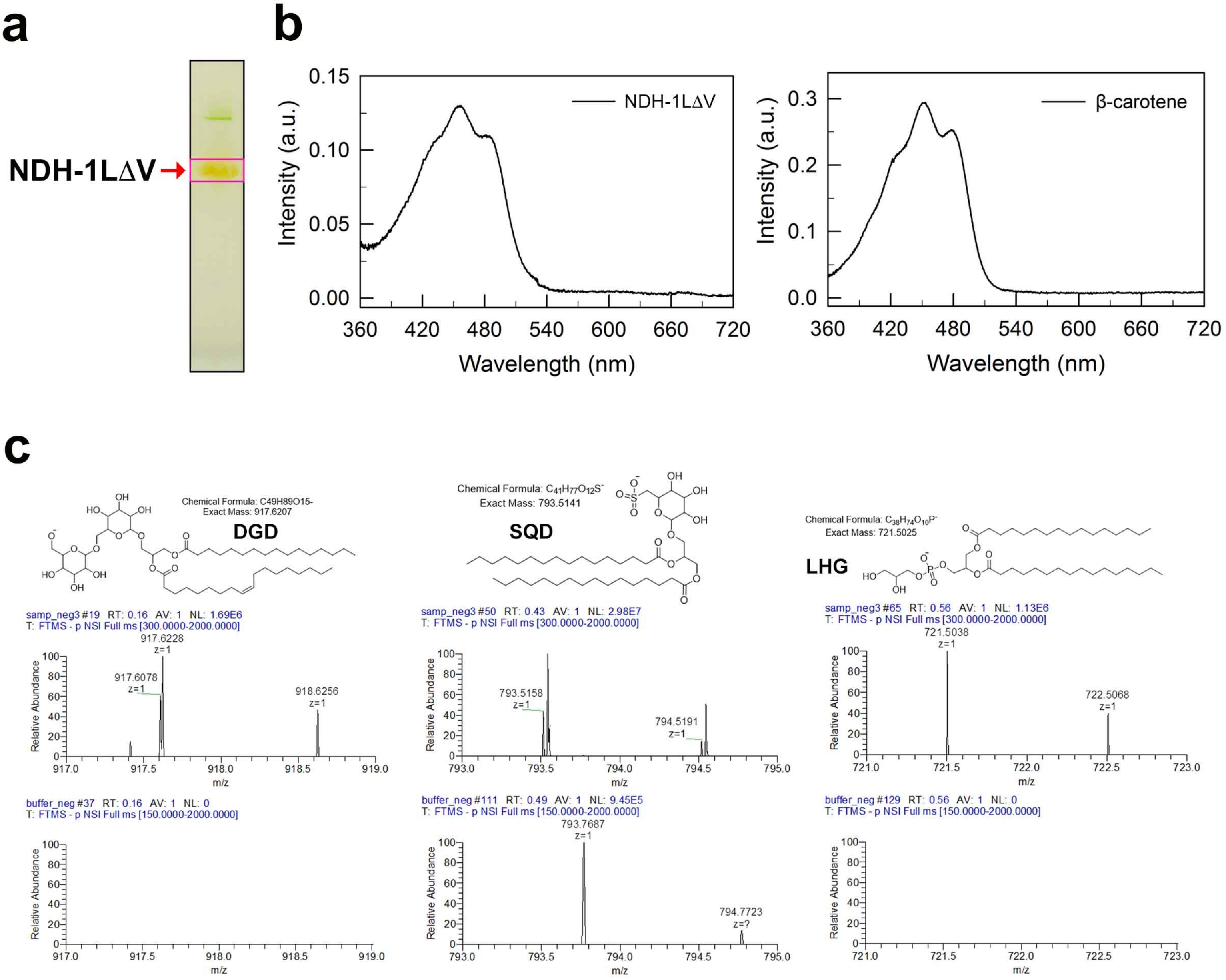

Supplementary Figure 8

Supplementary Figure 8

Identification of cofactors in purified NDH-1L complex

(a) Clear native (CN)-PAGE analysis of the purified NDH-1LΔV. **(b)** Absorption spectrum of NDH-1LΔV excised from the gel showed the characteristic absorption peaks of β-carotene. **(c)** Total lipids contained in purified NDH-1LΔV complex were extracted and analyzed by mass spectrometry. Three lipids, DGD, SQD and LHG could be determined in this analysis. Negative control was shown below.

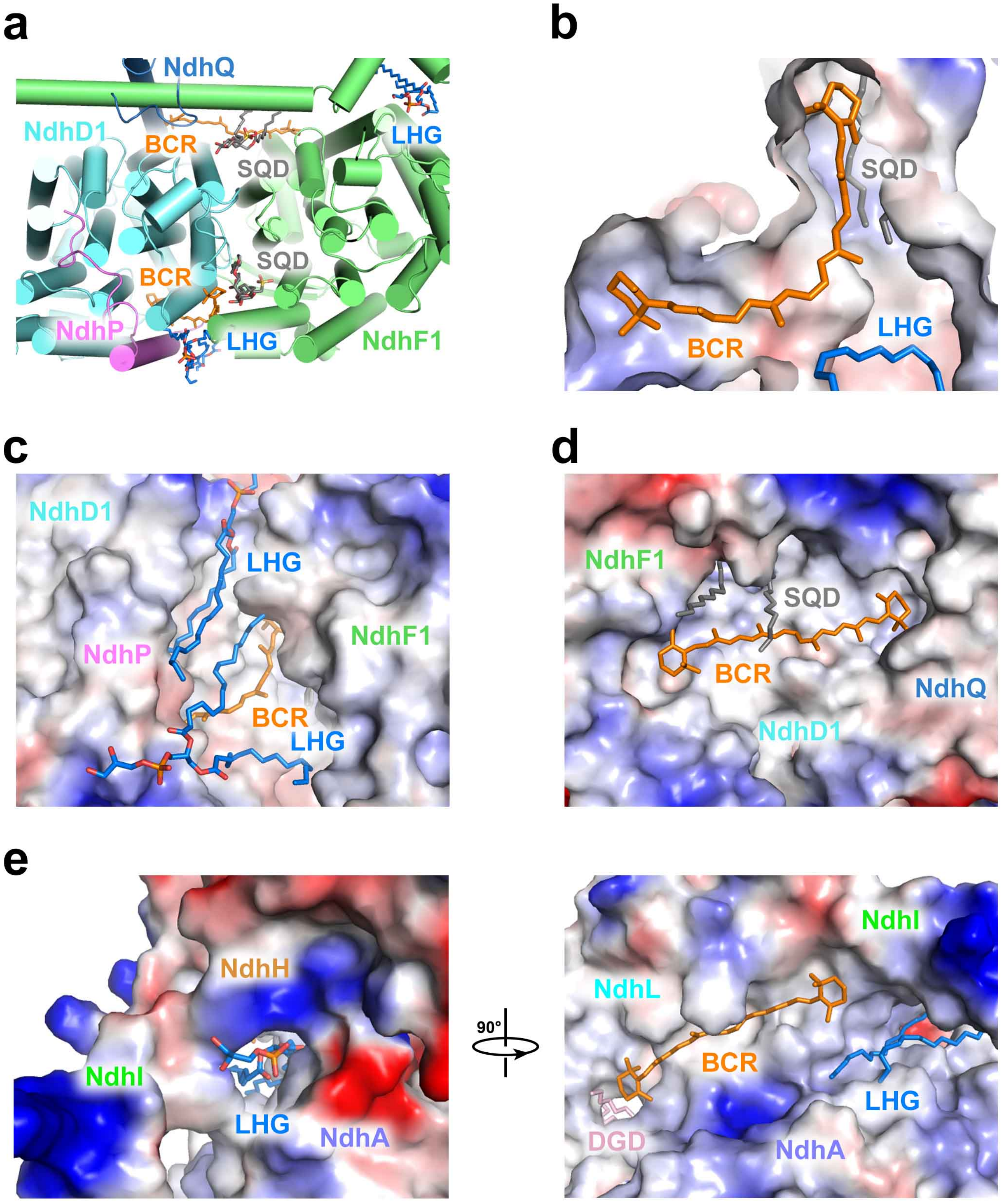

Supplementary Figure 9

Supplementary Figure 9

Close-up views of the interactions between β-carotene molecules and adjacent environments in the NDH-1L complex

(a) Top view of interactions between six cofactors located at the interface among NdhD1, NdhF1, NdhP and NdhQ. **(b)** The β-carotene molecule at the hydrophobic cavity between NdhD1 and NdhP. **(c)** The interaction between the β-carotene and two LHG molecules at the interface between NdhD1, NdhF1 and NdhP. **(d)** The β-carotene molecule on the hydrophobic surface between NdhD1, NdhF1 and NdhQ. **(e)** Interactions between the β-carotene, DGD and LHG at the hydrophobic cavity between NdhA, NdhI and NdhL. Subunits and cofactors of NDH-1L are color-coded and the scheme is shown as Fig. 1b and Fig. 2.

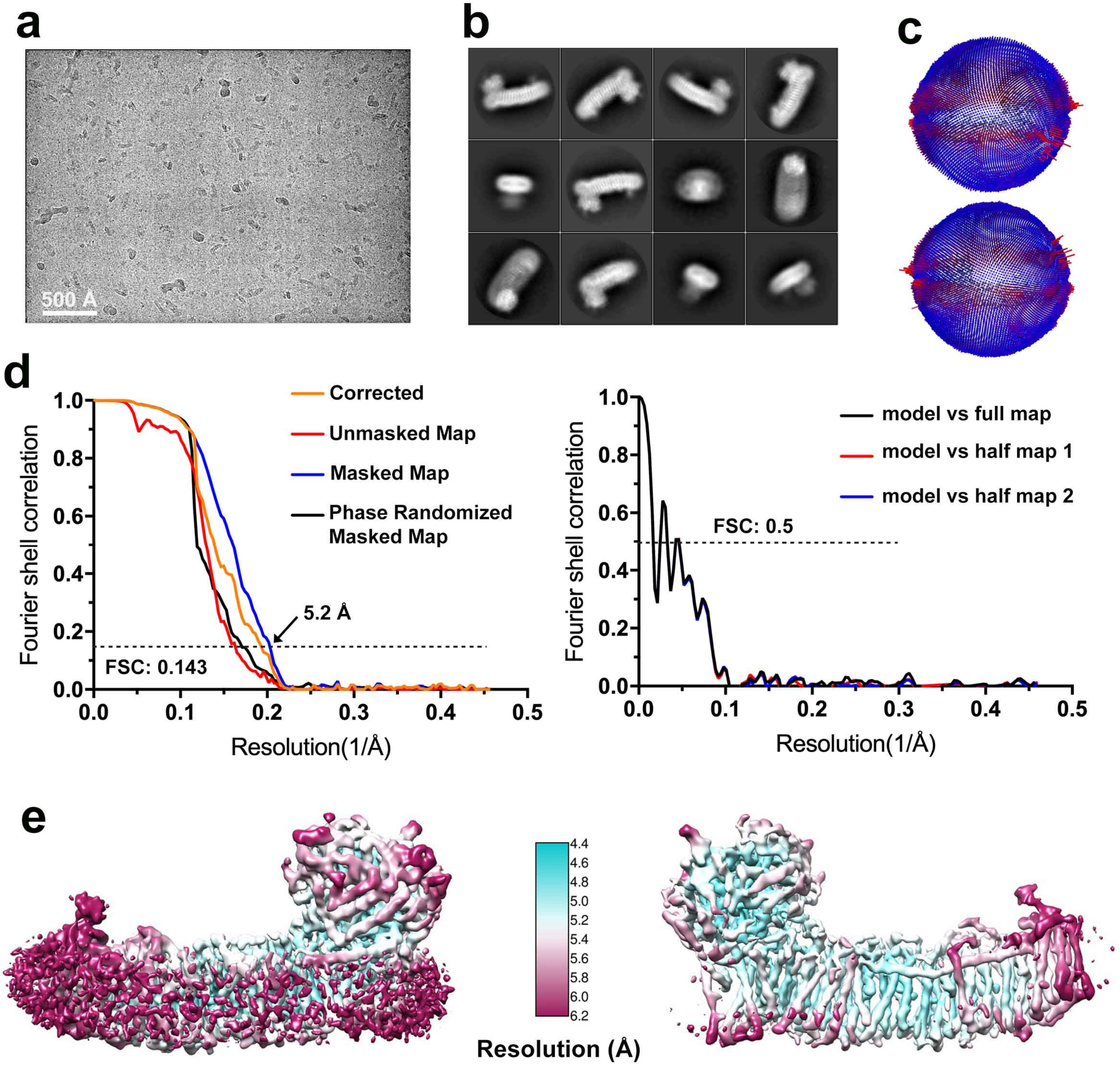

Supplementary Figure 10

Supplementary Figure 10

Cryo-EM analysis of the *T. elongatus* (Fd)-NDH-1LΔV complex

(a) A representative cryo-EM micrograph of the *T. elongatus* (Fd)-NDH-1LΔV complex. **(b)** Representative 2D class averages of (Fd)-NDH-1LΔV complex particles were obtained from reference-free classification. **(c)** Angular distributions of particles for the final reconstruction of the *T. elongatus* (Fd)-NDH-1LΔV complex. Red cylinders represent more particles on these orientations. Heights of cylinders indicate the relative numbers of particles. **(d)** The FSC curve of the final EM density map. The resolution was determined to an average of 5.2 Å with a cut-off value of 0.143 (left panel). The FSC curves were calculated between the refined atomic model and the half map used for refinement (red), the other half map (blue) and the full map (black) (right panel). The cut-off value of FSC is 0.5. **(e)** Local resolution of the final cryo-EM map estimated by RELION 3. The resolution is color-coded for different regions of the (Fd)-NDH-1LΔV complex. The left panel is drawn at a lower threshold. Extra densities in the membrane domain are those of the detergent (digitonin) used in the *in vitro* reconstitution of the (Fd)-NDH-1LΔV complex.

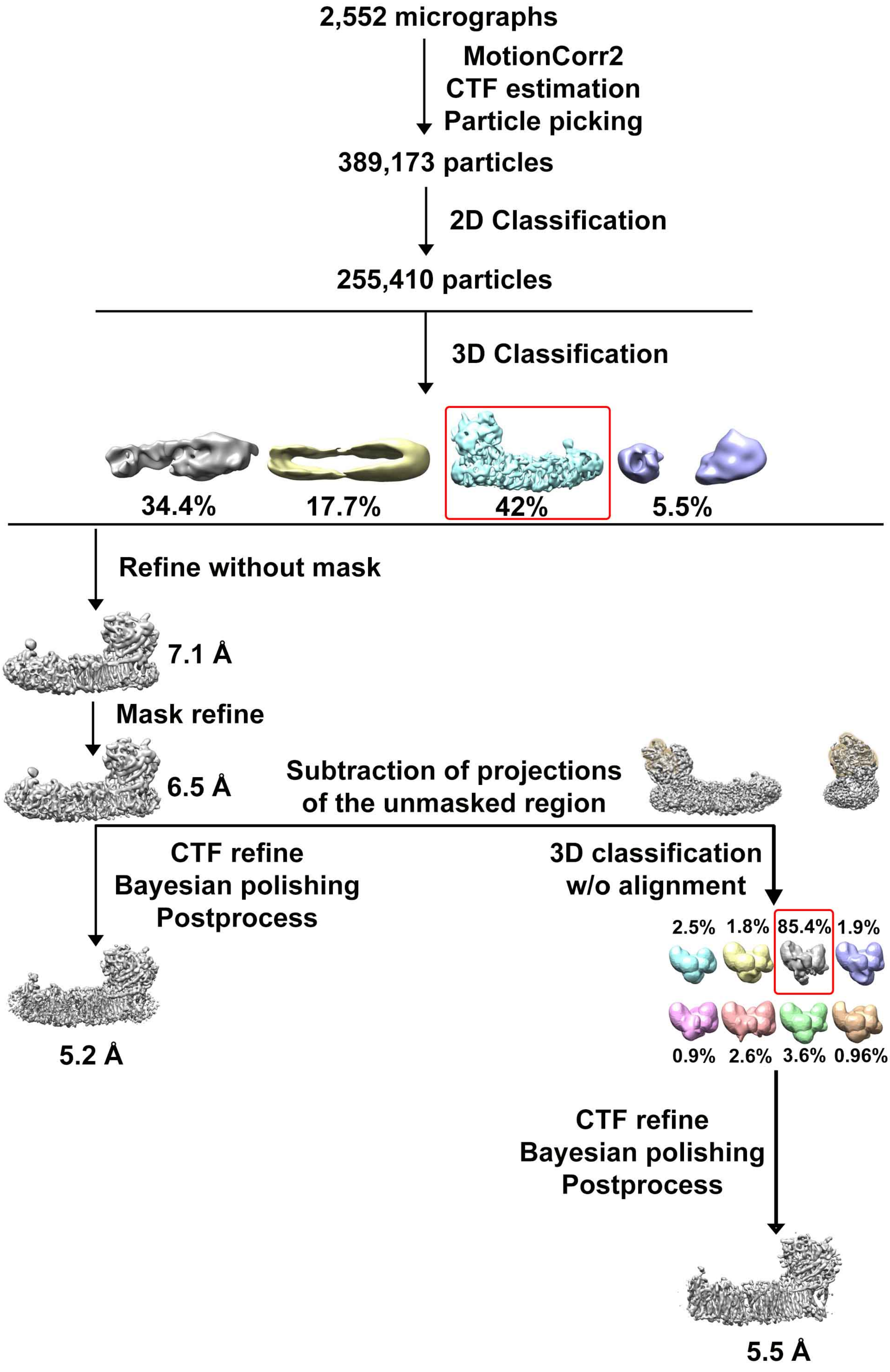

Supplementary Figure 11

Supplementary Figure 11

Flow chart for the cryo-EM data processing and structure determination of the *T. elongatus* (Fd)-NDH-1LΔV complex

Refer to Materials and Methods for details. The final reconstruction has an average resolution of 5.2 Å. All the images in this figure are created by Chimera.

a

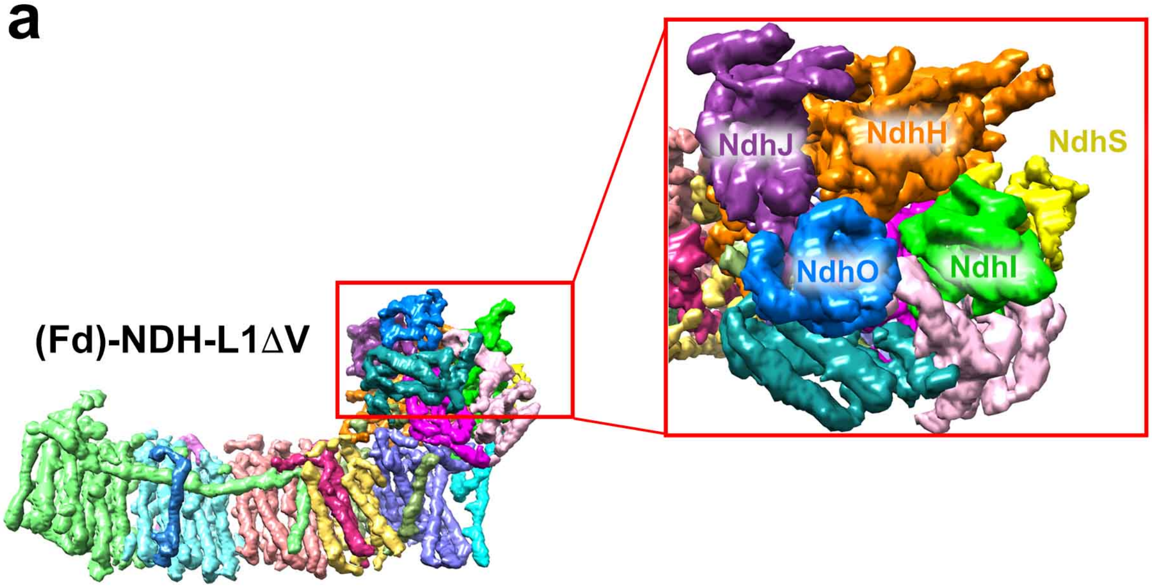

b

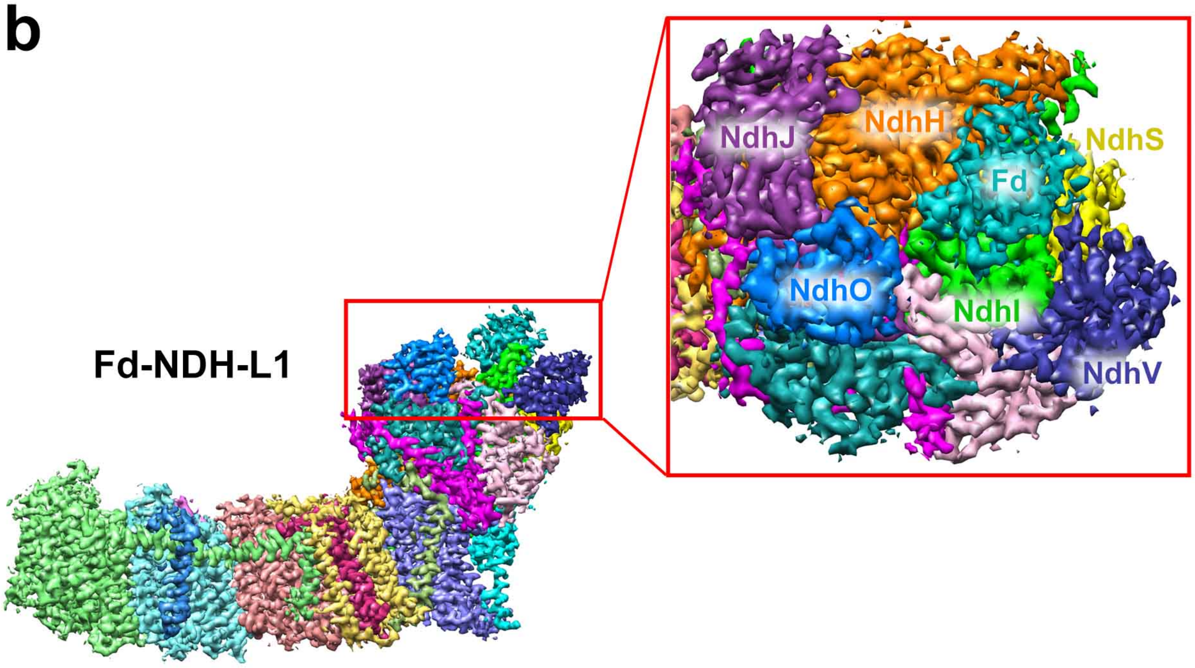

Supplementary Figure 12

Supplementary Figure 12

Comparison of Fd-binding pocket of Fd-NDH-1L complex with that of (Fd)-NDH-1LΔV complex

Fd is absent **(a)** or present **(b)** in the binding pocket of the NDH-1L complexes. Fd-binding pocket in the box region is enlarged in the inset shown on the right. Subunits of two complexes are colored as in **Fig. 1b**.

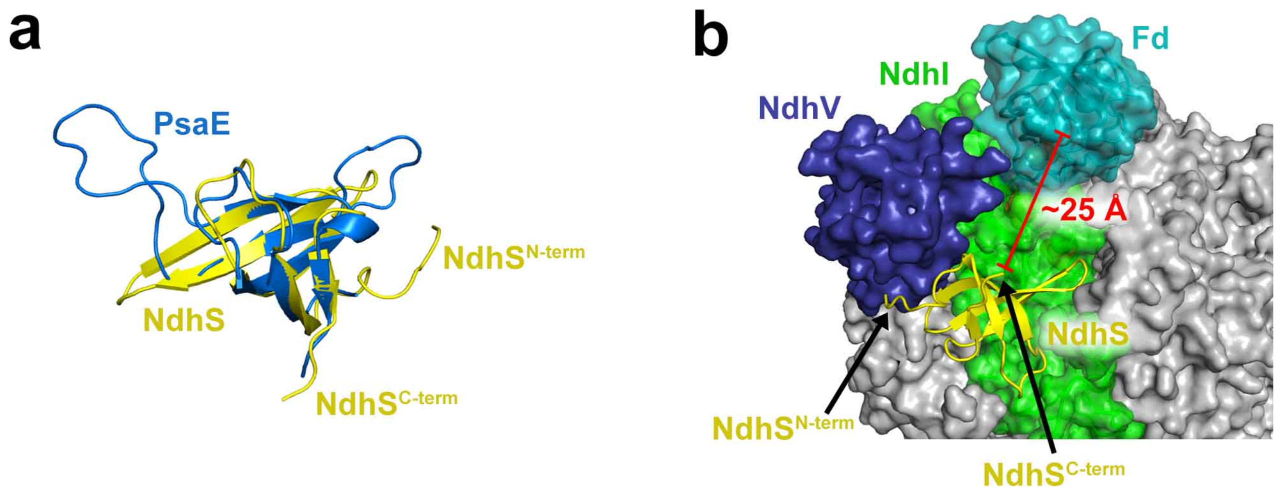

Supplementary Figure 13

Supplementary Figure 13

Structural analysis of the spatial organization of Fd, NdhS, NdhV and NdhI in the Fd-NDH-1L complex

(a) Superposition of NdhS and PsaE. Both proteins adopt an SH3-like fold. **(b)** NdhI (green), NdhV (purple) and Fd (cyan) are shown in surface representation and NdhS (yellow) is in cartoon. The distance from the C-terminus of NdhS to the nearest edge of Fd is about 25 Å.

Supplementary Table 1

Mass spectromety data of the purified NDH-1L complex

ORF	Protein name	Mascot score	Matched peptides	Coverage (%)
tlr0667	NdhA	504	16	11.0
tll0045	NdhB	734	26	9.0
tll0719	NdhD1	3630	78	20.2
tlr0670	NdhE	261	12	13.9
tll0720	NdhF1	510	22	8.8
tlr0669	NdhG	389	17	21.5
tlr1288	NdhH	3085	125	64.2
tlr0668	NdhI	4307	151	74.5
tlr1430	NdhJ	3984	98	83.9
tlr0705	NdhK	1939	65	32.9
tsr0706	NdhL	28	1	11.8
tll0447	NdhM	3447	62	49.6
tlr1130	NdhN	673	23	82.7
tsl0017	NdhO	739	23	87.1
/	NdhP	94	2	36.4
/	NdhQ	58	2	13.3
tlr0636	NdhS	408	8	41.8
tlr0472	NdhV	35	1	4.8

The yellow NDH-1L band is excised from the CN gel (Figure S8A) and is analyzed by mass spectrometry.

Supplementary Table 2

Cryo-EM data collection and refinement statistics

	Fd-NDH-1L (EMDB-0849) (PDB 6L7O)	NDH-1LΔV (EMDB-0850) (PDB 6L7P)	(Fd)-NDH-1LΔV (EMDB-0851)
**Data collection and processing**			
Magnification	75,000	75,000	81,000
Voltage (kV)	300	300	300
Electron exposure (e–/Å^2)	40	40	50
Defocus range (μm)	-1.8 to -2.7	-1.8 to -2.7	-1.5 to -3.0
Pixel size (Å)	1.09	1.09	1.09
Symmetry imposed	C1	C1	C1
Initial particle images (no.)	776,556	1,011,591	389,173
Final particle images (no.)	338,822	439,946	109,984
Map resolution (Å)	3.2	3.6	5.2
FSC threshold	0.143	0.143	0.143
Map resolution range (Å)	2.8-4.4	3.0-5.0	4.4-7.5
**Refinement**			
Initial model used (PDB code)	4HEA	4HEA	
Model resolution (Å)	3.3	3.3	
FSC threshold	n/a	n/a	
Model resolution range (Å)	n/a	n/a	
Map sharpening *B* factor (Å^2)	-94.7	-179	
Model composition			
Non-hydrogen atoms	33,116	31,389	
Protein residues	4,120	3,896	
Ligands	83	66	
B factors (Å^2)			
Protein	65.47	44.93	
Ligand	67.12	47.13	
R.m.s. deviations			
Bond lengths (Å)	0.008	0.009	
Bond angles (°)	1.566	1.696	
Validation			
MolProbity score	1.76	1.58	
Clashscore	7.28	3.58	
Poor rotamers (%)	0.00	0.22	
Ramachandran plot			
Favored (%)	94.73	93.38	
Allowed (%)	5.27	6.59	
Disallowed (%)	0.00	0.22	

Supplementary Table 3

Oligonucleotides used for quantitative RT-PCR

Primer Name	Description
16s-F	AGAACCTACCTTCAGAATG
16s-R	CCATCTTCAGACGATAAATC
NdhA-F	GGAAGCCGAAGAAGAATT
NdhA-R	TAGGAGCCAAGGTAGAAC
NdhB-F	TTGTCATTGCTGGTATTG
NdhB-R	GTGGGAGAACCTTCATAA
NdhC-F	TTCAACATCCGCTACTAC
NdhC-R	TGCTTCTACAAAGGCTAA
NdhD1-F	ATCGCCGCTAAGGACATT
NdhD1-R	GCAACTTAACGCCGTAGG
NdhD3-F	TCTTAATAACCTGTTGAC
NdhD3-R	GACGATGAATAGAAGTAG
NdhE-F	TCATCACTAGCCGTAATG
NdhE-R	CCATCAGGTTGAGGTTAA
NdhF1-F	CTGTTGGTGATGATCTAC
NdhF1-R	AGACTGAGATAGGCATAG
NdhF3-F	TAGGTACTTATCTTCTCA
NdhF3-R	CATCATAATTCCAACTTC
NdhG-F	AGGTGTTCAGTACATATC
NdhG-R	CAATAGGAAGGCAGAATA
NdhH-F	TAAGGTGGATCATTACGA
NdhH-R	AATTCTGACTAGGTAACG
NdhI-F	AATGTATTGCCTGTGAAG
NdhI-R	CGATGCTGTAATGTTTGA
NdhJ-F	GCCTATGGGTTCAACTATT
NdhJ-R	TATCCTCGGTCAACTTCA
NdhK-F	CGGTGGATGTGTATATTC
NdhK-R	TCTTGGATAGATTCATTGG
NdhL-F	CTAAGCCTAGCCTATCTAT
NdhL-R	GAAGAAGAGAAAGACCAAA
NdhM-F	CAAGGTAATGAACTGATTCC
NdhM-R	CTGTTGGAGAGCATCTTC
NdhN-F	CATTGCCATTAATTGCTA
NdhN-R	CTTCATAACCACCTTCTA
NdhO-F	AGTTTAGTGCGGGTTATC
NdhO-R	ACTTCTCCTTTGCTATGG
NdhP-F	GTTTACCCTGGCATGTCTA
NdhP-R	CAGAACCATTACCGTCGTA
NdhQ-F	CTGAACCGTGGCATCATG
NdhQ-R	AGATAATCAGGGCACCAATG
NdhS-F	TGCGAGTCACCAATGTTG
NdhS-R	CAGCCTTACCGTCACTAAC
NdhV-F	TTCAACCTCTACTTCTTC
NdhV-R	TCAATCATAAAGGACTCTA
CupA-F	GATACCTACCTAGATAGTGA
CupA-R	ATAGTTCTCTGACCTGTT

CupS-F	ATGTCGCCTATCAACTGT
CupS-R	ATGATTACCGTGGCATTT
