# Supplementary material for: Structural insights into NDH-1 mediated cyclic electron transfer
Source: Nat Commun. 2020 Feb 14;11:888. doi: 10.1038/s41467-020-14732-z (PMC7021789; doi:10.1038/s41467-020-14732-z)
Supplement: Supplementary file 3 — Reporting Summary [file 41467_2020_14732_MOESM3_ESM.pdf]

nature research

Corresponding author(s): Ming Lei

Last updated by author(s): Jan 17, 2020

Reporting Summary

Nature Research wishes to improve the reproducibility of the work that we publish. This form provides structure for consistency and transparency in reporting. For further information on Nature Research policies, see Authors & Referees and the Editorial Policy Checklist .

Statistics

For all statistical analyses, confirm that the following items are present in the figure legend, table legend, main text, or Methods section.

n/a	Confirmed	
☐	☒	The exact sample size (*n*) for each experimental group/condition, given as a discrete number and unit of measurement
☐	☒	A statement on whether measurements were taken from distinct samples or whether the same sample was measured repeatedly
☒	☐	The statistical test(s) used AND whether they are one- or two-sided<br>*Only common tests should be described solely by name; describe more complex techniques in the Methods section.*
☒	☐	A description of all covariates tested
☒	☐	A description of any assumptions or corrections, such as tests of normality and adjustment for multiple comparisons
☒	☐	A full description of the statistical parameters including central tendency (e.g. means) or other basic estimates (e.g. regression coefficient) AND variation (e.g. standard deviation) or associated estimates of uncertainty (e.g. confidence intervals)
☒	☐	For null hypothesis testing, the test statistic (e.g. *F*, *t*, *r*) with confidence intervals, effect sizes, degrees of freedom and *P* value noted<br>*Give P values as exact values whenever suitable.*
☒	☐	For Bayesian analysis, information on the choice of priors and Markov chain Monte Carlo settings
☒	☐	For hierarchical and complex designs, identification of the appropriate level for tests and full reporting of outcomes
☒	☐	Estimates of effect sizes (e.g. Cohen's *d*, Pearson's *r*), indicating how they were calculated

Our web collection on statistics for biologists contains articles on many of the points above.

Software and code

Policy information about availability of computer code

Data collection	EPU
Data analysis	Gctf_v1.06, MotionCor2-v1.0.0, RELION-v3.0.b2, Gautomatch-v0.56, Chimera-v1.11.2, COOT-v0.89, PHENIX-v1.14, PsiPred, CAVER 3.0, PyMOL, Prism 7, MolProbity, MASCOT-v2.2.

For manuscripts utilizing custom algorithms or software that are central to the research but not yet described in published literature, software must be made available to editors/reviewers. We strongly encourage code deposition in a community repository (e.g. GitHub). See the Nature Research guidelines for submitting code & software for further information.

Data

Policy information about availability of data

All manuscripts must include a data availability statement. This statement should provide the following information, where applicable:

- Accession codes, unique identifiers, or web links for publicly available datasets
- A list of figures that have associated raw data
- A description of any restrictions on data availability

We have provided a full data availability statement in the manuscript.

Field-specific reporting

Please select the one below that is the best fit for your research. If you are not sure, read the appropriate sections before making your selection.

☒ Life sciences ☐ Behavioural & social sciences ☐ Ecological, evolutionary & environmental sciences

For a reference copy of the document with all sections, see nature.com/documents/nr-reporting-summary-flat.pdf

Life sciences study design

All studies must disclose on these points even when the disclosure is negative.

Sample size	Cryo-EM datasets for NDH-1LΔV and Fd-NDH-1L were collected during two 10-day sessions, resulting in 439,946 and 338,822 particles for final reconstitution. Dataset for (Fd)-NDH-1LΔV was collected during 2 days sessions, resulting in 109,984 particles for final reconstitution.
Data exclusions	Low-quality and false-positive particles were excluded during 2D and 3D classifications, as a standard image processing practice in cryo-EM.
Replication	All attempts to replication were successful.
Randomization	N/A
Blinding	N/A

Reporting for specific materials, systems and methods

We require information from authors about some types of materials, experimental systems and methods used in many studies. Here, indicate whether each material, system or method listed is relevant to your study. If you are not sure if a list item applies to your research, read the appropriate section before selecting a response.

Materials & experimental systems

n/a	Involved in the study
☐	☒ Antibodies
☒	☐ Eukaryotic cell lines
☒	☐ Palaeontology
☒	☐ Animals and other organisms
☒	☐ Human research participants
☒	☐ Clinical data

Methods

n/a	Involved in the study
☒	☐ ChIP-seq
☒	☐ Flow cytometry
☒	☐ MRI-based neuroimaging

Antibodies

Antibodies used	The following antibodies were used: antibody against NdhH, NdhI, NdhJ, NdhK, NdhM, NdhO, NdhV and ATPβ were previously raised in our lab. Antibody against CupA was provided from Professor Hualing Mi (Institute of Plant Physiology and Ecology, Chinese Academy of Sciences) lab.
Validation	All these antibodies were prepared from the cyanobacterium Synechocystis sp. strain PCC 6803 and were validated based on positive and negative control cell strains.
